# Feasibility of diffusion-weighted magnetic resonance imaging in evaluation of early therapeutic response after CT-guided microwave ablation of inoperable lung neoplasms

**DOI:** 10.1007/s00330-021-08387-7

**Published:** 2021-11-19

**Authors:** Thomas J. Vogl, Emad H. Emara, Elsayed Elhawash, Nagy N. N. Naguib, Mona O. Aboelezz, Hossam M. Abdelrahman, Sameh Saber, Nour-Eldin A. Nour-Eldin

**Affiliations:** 1 Institute for Diagnostic and Interventional Radiology, Johan Wolfgang Goethe – University Hospital, Theodor-Stern-Kai 7, 60590 Frankfurt am Main, Germany; 2 Department of Diagnostic and Interventional Radiology, Kafrelsheikh University, Kafr Elsheikh, Egypt; 3 Department of Diagnostic and Interventional Radiology, University of Alexandria, Alexandria, Egypt; 4 Department of Radiology, University of Zagazig, Zagazig, Egypt; 5 Department of Diagnostic and Interventional Radiology, Cairo University Hospital, Cairo, Egypt

**Keywords:** Microwave ablation, Diffusion magnetic resonance imaging, Treatment outcome, Lung neoplasms

## Abstract

**Objective:**

To determine the early treatment response after microwave ablation (MWA) of inoperable lung neoplasms using the apparent diffusion coefficient (ADC) value calculated 24 h after the ablation.

**Materials and methods:**

This retrospective study included 47 patients with 68 lung lesions, who underwent percutaneous MWA from January 2008 to December 2017. Evaluation of the lesions was done using MRI including DWI sequence with ADC value calculation pre-ablation and 24 h post-ablation. DWI-MR was performed with *b* values (50, 400, 800 mm^2^/s). The post-ablation follow-up was performed using chest CT and/or MRI within 24 h following the procedure; after 3, 6, 9, and 12 months; and every 6 months onwards to determine the local tumor response. The post-ablation ADC value changes were compared to the end response of the lesions.

**Results:**

Forty-seven patients (mean age: 63.8 ± 14.2 years, 25 women) with 68 lesions having a mean tumor size of 1.5 ± 0.9 cm (range: 0.7–5 cm) were evaluated. Sixty-one lesions (89.7%) showed a complete treatment response, and the remaining 7 lesions (10.3%) showed a local progression (residual activity). There was a statistically significant difference regarding the ADC value measured 24 h after the ablation between the responding (1.7 ± 0.3 × 10^−3^ mm^2^/s) and non-responding groups (1.4 ± 0.3 × 10^−3^ mm^2^/s) with significantly higher values in the responding group (*p* = 0.001). A suggested ADC cut-off value of 1.42 could be used as a reference point for the post-ablation response prediction (sensitivity: 66.67%, specificity: 84.21%, PPV: 66.7%, and NPV: 84.2%). No significant difference was reported regarding the ADC value performed before the ablation as a factor for the prognosis of treatment response (*p* = 0.86).

**Conclusion:**

ADC value assessment following ablation may allow the early prediction of treatment efficacy after MWA of inoperable lung neoplasms.

**Key Points:**

*• ADC value calculated 24 h post-treatment may allow the early prediction of MWA efficacy as a treatment of pulmonary tumors and can be used in the early immediate post-ablation imaging follow-up.*

*• The pre-treatment ADC value of lung neoplasms is not different between the responding and non-responding tumors.*

## Introduction

Percutaneous local tumor ablation under image guidance is a minimally invasive therapeutic option for the management of inoperable primary and metastatic lung tumors, using different ablative tools including radiofrequency (RF), microwave (MW), and cryoablation. The rate of local tumor residual and/or progression after the ablation may reach up to 30%. Therefore, early post-ablation detection of local tumor residual or progression is of extreme value to allow early interference in such cases [1–3]. The early definition of tumor response after ablation using the CT scan is limited by two main interfering factors. Firstly, the ablated lesion in the early CT scan apparently looks larger than the pre-ablation size as a part of the normal lung parenchyma surrounding the lesion is ablated within the context of safety margin giving a larger ablation zone. This zone of ablation subsequently shrinks over time [4, 5]. The second limiting factor is the region of ground-glass opacity surrounding the ablated lesion which may be accompanied by a parenchymal lung hemorrhage due to the mechanical injury of lung parenchyma during the ablation procedure [6].

Diffusion-weighted magnetic resonance imaging (DW-MRI) is a functional imaging sequence that depends on the measurement of the random movement of water molecules in tissues at a microscopic level [7, 8]. Many studies revealed the usefulness of DW-MRI in the evaluation of treatment response after local thermal ablation of tumors such as hepatocellular carcinoma (HCC), lung tumors, and implanted tumors in mice and rabbits [8–12] as an early predictor for treatment response after local tumor ablation, and some of these studies showed significantly higher apparent diffusion coefficient (ADC) values early within days after ablation in properly ablated lesions compared to lower values in residual tumor lesions [8, 10].

A study by Okuma et al [10] addressed the role of the ADC value in predicting the early treatment response after lung radiofrequency ablation on a relatively small number of patients; besides, no post-ablation cut-off value to predict response was suggested. To our knowledge, no other study addressed the subject of diffusion-weighted imaging immediately after microwave ablation of malignant pulmonary lesions. Based on this, we performed the current study on a relatively large number of patients to evaluate the role of diffusion-weighted imaging and ADC value measurement in the ablated pulmonary lesions in MRI studies performed 24 h after microwave ablation as an early predicting tool for the local treatment response. Also, evaluation of the lung tumor ablation by CT volumetry was not possible within 3 months post-ablation [13].

The purpose of the current study was to evaluate the role of ADC value measurement in the ablated pulmonary lesions in MRI studies performed 24 h after microwave ablation as an early predicting tool for the local treatment response.

## Materials and methods

This retrospective study included 47 patients; all underwent microwave ablation (MWA) of 68 lesions in 68 treatment sessions from January 2008 to December 2017. The study protocol was approved by our university hospital ethical committee board. All patients provided signed informed consent for MWA and follow-up imaging protocols including the use of clinical data for research purposes. All patients received DWI-MRI imaging protocol 48-hours pre-ablation and 24-hours post-ablation for tumor assessment. The demographic data of the study group and histopathology of the lesions are summarized in (Table 1).

**Table 1:** Patients’ demographic data

Criteria	Patients’ data
Number of patients	47
Number of lesions	68
Sex (man:woman)	22:25
Age (years), mean/SD	63.8 ± 14.2 (range 30–83 years)
Site of lesion (right:left)	38:30
Lesion location (central:peripheral)	9:59
Tumor size before ablation	0.7-5 cm
Pre-ablation treatment	
Systemic chemotherapy	30
Radiotherapy	0
Pulmonary resection	3
Pathology of lung lesions	**Number of patients**
Bronchogenic carcinoma	7
Metastatic colorectal carcinoma	19
Metastatic breast carcinoma	8
Metastatic parotid adenocarcinoma	3
Metastatic renal cell carcinoma	4
Metastatic hepatocellular carcinoma	4
Metastatic endometrial carcinoma	2

The inclusion criteria for ablation therapy were as follows: (a) patients had to have surgically unresectable pulmonary metastases, (b) patients had to be poor candidates for surgery because of medical reasons including limited cardiopulmonary reserve, (c) patients had to have metastases after pneumonectomy or recurrent metastases after surgical resection, (d) patients had to have five or fewer lesions, and (e) the lesions had to be 5 cm or smaller in maximal axial diameter. The exclusion criteria were as follows: (a) the primary malignancy was uncontrolled, (b) there was extrathoracic spread, (c) there were more than five lesions, (d) the lesions were larger than 5 cm in maximal axial diameter, (e) there was radiologic evidence of lymph node metastases, (f) there were tumors infiltrating the chest wall or mediastinal structures, (g) there was uncorrectable coagulopathy (as indicated by an international normalized ratio > 1.8 or a platelet count < 50,000), (h) there was septicemia, and/or (i) the patient refused ablation therapy.

### Pre-ablation assessment

The decision for ablation therapy was justified by the thoracic multidisciplinary tumor board including an interventional radiologist, thoracic surgeons, and pulmonology and medical oncology physicians. The clinical data and all imaging studies of the patients were thoroughly reviewed. The indications, possible risks, complications, and benefits of the technique were discussed case by case. Pre-interventional laboratory parameters including complete blood count and coagulation profile were assessed. Anticoagulant therapy or antiplatelet medications were stopped 1 week before the ablation to avoid the potential risk of bleeding.

### MWA procedure

A planned chest CT scan was carried out before the treatment to confirm the number, size, and site of the targeted lesions as well as the condition of the lung. The parameters of the ablation procedures were determined depending on the lesion size and location, including the length and number of the MW antennae, the positioning of the patient, and the access of the ablation track. The patient position was adjusted depending on the site of the lesion to accomplish the shortest and safest track with good accessibility to the lesion in the most comfortable position to the patient.

The ablations were done under the guidance of CT (Somatom Sensation 64 slices; Siemens) with the following scanning parameters: kV: 120, mAs: 30, 5-mm section collimation, and 5-mm slice thickness. All MWA procedures were performed by two radiologists specialized in the interventional procedures with experience of more than 9 and 15 years in thoracic interventions under complete aseptic conditions. Procedures were accomplished under conscious sedation using 5 mg piritramide and 5 mg midazolam with continuous monitoring of vital signs through the procedure.

The microwave ablation procedures were done using antennae (12, 17, or 22 cm shaft length and 3.7 cm radiating section) with microwave generators (Tyco Healthcare Group) of power settings (35–45 W) with 15 min mean time of ablation (range: 10 to 30 min). The ablation times were recorded.

### Follow-up imaging protocol

#### MRI

MRI was performed 2 days before the ablation procedure as a baseline study and 24 h after MWA using a 1.5-T MRI machine (Avanto-Fit, Siemens Healthcare). MRI sequences included transaxial pre- and post-contrast T1-weighted sequences (T1 WI) with breath-holding (TR/TE: 6.69/4.77 ms, slice thickness: 5 mm, field of view (FOV): 325 × 400 mm^2^, matrix size: 256 × 208), transaxial breath-holding T2-weighted image (T2 WI) with fat saturation (TR/TE: 1000/90 ms, slice thickness: 5 mm, FOV: 310 × 400 mm^2^, matrix size: 320 × 186), coronal T2 WI with breath-holding (TR/TE: 1200/99, slice thickness: 5 mm, FOV: 430 × 430 mm^2^, matrix size: 256 × 256), and quiet breathing short-time inversion recovery (SPAIR)-DWI (TR/TE: 5700/82 ms; *b* values 50, 400, and 800 mm^2^/s; acquisition matrix = 128 × 128, 6 mm slice thickness, gapless).

#### CT

CT of the chest with contrast (64 slices; Siemens, using the following scanning parameters: Kv: 120, mAs: 30, section collimation: 5 mm, and slice thickness: 5 mm) within 24 h and in 3, 6, 9, and 12 months post-microwave ablation procedure, then every 6 months onwards to determine the response and local tumor progression. The treatment outcome after local thermal ablation was judged by CT scan series taken 3 months following MWA or later.

### Imaging assessment

Two radiologists with experience of more than 9 and 15 years in thoracic radiology assessed the MRI and CT images and calculated the ADC values using ADC maps generated from the SPAIR-DWI sequence in consensus. Axial T1 WI, T2 WI, and DW images were graded as hypo-, iso-, or hyperintense as compared to muscles. MRI data reconstruction was done using the Siemens workstation (SYNGO), and apparent diffusion coefficient maps were then generated by available software and the imaging workstation. The region of interest (ROI) was placed at most of the tumor mass to calculate ADC values using the single-slice technique. The ROI was defined by tracing a line along the perceived tumor margins on DWI (median ROI area: 231 mm^2^, range: 35–984 mm^2^). During the evaluation of treatment outcome after local ablation using CT chest examination, a comparison of the follow-up CT images with the images that were immediately done after the ablation procedure was performed. Response to treatment was considered when the ablated lesion decreased in size over time with no contrast uptake. The contrast enhancement pattern was the determining factor of success or failure of tumor ablation. Irregular focal soft-tissue enhancement (> 15 HU) was considered to be a sign of residual or recurrent disease. A thin symmetric rim of peripheral enhancement of less than 5 mm in width observed up to 6 months after ablation was considered a sign of benign perilesional enhancement. This was based on the parameters used by different scientific institutions in previous studies [11, 12]. The CT and MRI images pre- and post-procedure were then precisely reviewed and compared to the MRI images obtained immediately after ablation, and a comparison of changes in the mean ADC values immediately after MWA with the end tumor local response was done relying on the chest CT and/or MRI imaging during the follow-up. The image evaluation was performed in chronologically separate sessions where the readers first evaluated the ADC value changes and were blinded to the patient local response at the follow-up, and in the second reading session, the cases were presented in a random sequence and the authors did not have access to the MRI images and ADC values from the first reading session.

### Statistical analysis of the data

The study patients were assigned into two groups based on the treatment response with subsequent follow-up, the responsive group and the local progression group with residual tumoral activity. Analysis of the data was done using IBM SPSS software version 20.0. The Kolmogorov-Smirnov test was used to verify the normality of the distribution of variables. The Student *t* test was used to compare the two groups for normally distributed quantitative variables. The diagnostic performance of the markers was determined by the receiver operating characteristic curve (ROC). The acceptable performance of the test is given with an area of more than 50% while the area of about 100% gives the best performance. The judgment of the significance of the obtained results was at the 5% level (*p* value).

## Results

Forty-seven patients with a mean age of 63.8 ± 14.2 years (range 30–83 years, 25 women and 22 men) having 68 lung lesions were included. The most common lung lesions were of colorectal origin (36.7%) followed by metastatic breast carcinoma (19.1%) (Table 1).

All the lesions showed a complete ablation zone with an adequate safety margin at ablation. Sixty-one lesions (89.7%) had shown a complete treatment response while seven lesions (10.3%) showed a residual activity (incomplete response) based on the follow-up periods ranging from 6 to 36 months.

The mean tumor size was 1.5 ± 0.9 cm (range: 0.7–5 cm), and the mean tumor volume was 2.2 cm^3^ (range: 0.1–61.3 cm^3^). There was a statistically significant difference in pre-ablation tumor size between the responding and local progression groups (*p* = 0.018) with a larger size of lesions in the local progression group.

**Fig. 1:**
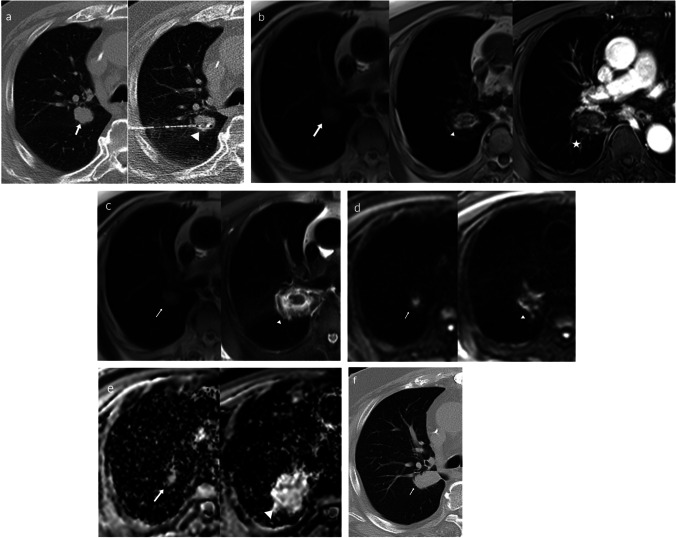
A 75-year-old male patient with metachronous pulmonary metastasis from colorectal cancer. **a** Combined CT images showing the pre-ablation right perihilar metastatic lesion (left-image arrow) and CT image during MWA (arrowhead). **b** Combined MRI images in T1WI pre-ablation (left image), T1WI 24 h post-ablation (middle image), and T1WI with contrast 24 h post-ablation (right image) displaying low signal intensity before and after ablation with marginal contrast enhancement after ablation. **c** Combined MRI images in T2WI pre-ablation (left image) and T2WI 24 h post-ablation (right image) displaying high signal before ablation and central low signal intensity with marginal high signal after ablation. **d** DWI pre-ablation (left image) and DWI 24 h post-ablation (right image) displaying high signal intensity before ablation with reduction of the signal intensity after ablation. **e** ADC map pre-ablation (left image) and ADC map 24 h post-ablation (right image). **f** In the CT follow-up after 6 months, the lesion shows a fibrotic band with no contrast enhancement denoting local tumor response

**Fig. 2:**
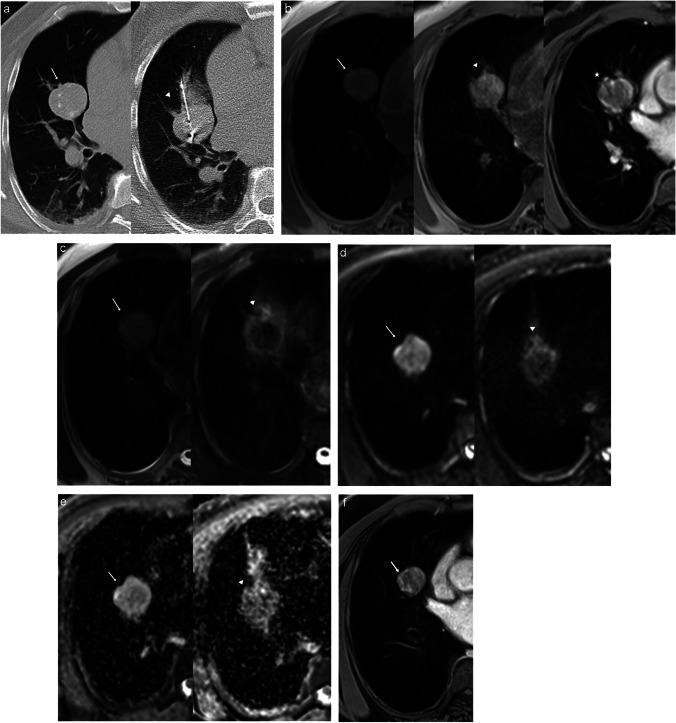
A 50-year-old male patient with renal cell carcinoma. The patient developed metachronous right pulmonary metastasis. **a** Combined CT images showing the pre-ablation metastatic lesion (left-image arrow) and during MWA (arrowhead). **b** Combined MRI images in T1WI pre-ablation (left image), T1WI 24 h post-ablation (middle image), and T1WI with contrast 24 h post-ablation (right image) displaying low signal intensity before and after ablation with irregular thick marginal contrast enhancement after ablation. **c** Combined MRI images in T2WI pre-ablation (left image) and T2WI 24 h post-ablation (right image) displaying high signal before ablation and central low signal intensity with marginal high signal after ablation. **d** DWI pre-ablation (left image) and DWI 24 h post-ablation (right image) displaying high signal intensity before ablation with reduction of the high signal intensity after ablation. **e** ADC map pre-ablation (left image) and ADC map 24 h post-ablation (right image). **f** T1WI post-contrast follow-up after 6 months showing nodular contrast enhancement of the lesion denoting residual local tumor activity

All lesions in the MRI study before ablation demonstrated low signal intensity at T1 WI, high signal intensity at T2 WI, and high signal at DWI. In the MRI study performed 24 h post-ablation, the lesions elicited low signal intensity in the center of the ablation zone with a high marginal signal intensity in the periphery of the ablation zone in T2 WI with marginal enhancement in post-contrast T1 WI (Figs. 1 and 2).

Before treatment, there was no significant difference in the mean ADC values of both responding (0.8 ± 0.2 × 10^−3^ mm^2^/s) and local tumor progression groups (0.7 ± 0.2 × 10^−3^ mm^2^/s, *p* = 0.857). However, the post-ablation mean ADC values in lesions that responded to local ablation were significantly higher in comparison with those in non-responding lesions with local tumor progression (*p* = 0.001) (Table 2).

**Table 2:** The relation between ADC value and local tumor response

	Complete response (*n* = 61)	Local progression/residual (*n* = 7)	*p* value
ADC before ablation	0.8 ± 0.3 × 10^−3^ mm^2^/s	0.7 ± 0.3 × 10^−3^ mm^2^/s	0.857
ADC (24 h) after ablation	1.7 ± 0.2 × 10^−3^ mm^2^/s	1.4 ± 0.2 × 10^−3^ mm^2^/s	0.001

**Fig. 3:**
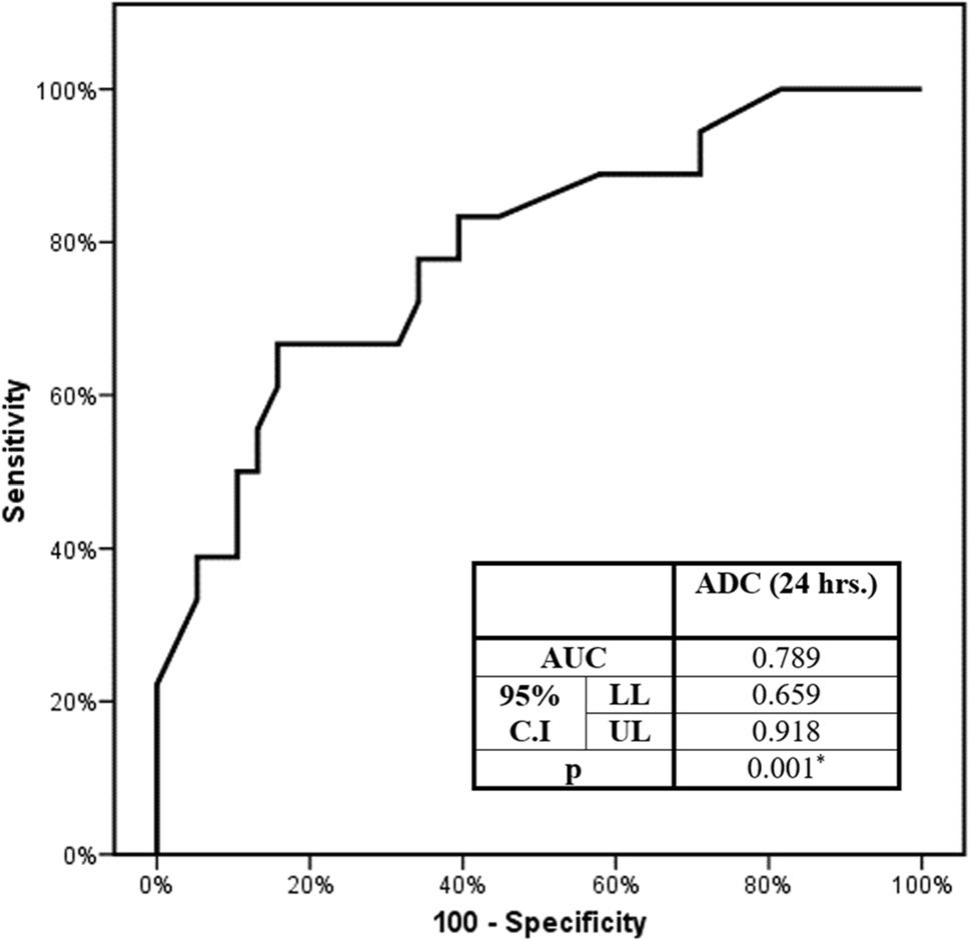
Receiver operating curve (ROC) for ADC (24 h) diagnoses an incomplete response. AUC, area under the curve; C.I, confidence interval; LL, lower limit; UL, upper limit

A suggested cut-off ADC value of 1.42 has been used as a reference point for the prediction of the local tumor response after MWA (sensitivity: 66.67%, specificity: 84.21%, PPV: 66.7%, and NPV: 84.2%) (Fig. 3).

There was no statistically significant correlation between tumor size before the ablation and pre-ablation ADC values of both responsive and local progression lesions (*p* = 0.263 and *p* = 0.432, respectively) and post-ablation ADC values of lesions with local progression (*p* = 0.317) as well. But a significant positive correlation was noticed between tumor size before the ablation and post-ablation ADC values of responsive lesions (*p* = 0.031).

## Discussion

The current advances in functional imaging procedures offered the ability to depict microscopic changes in tumor structure and environment, hence allowing better evaluation of the response following local thermal ablation of the tumors through observing changes in cell viability, tissue perfusion, and vascularity of the tumor. CT examination has long been used for the assessment of tumor ablation response after local ablation. However, there is growing evidence that supports the usage of MRI for this purpose. Diffusion-weighted MR imaging with ADC value calculation is a useful diagnostic imaging tool in the follow-up after local thermal ablation and can detect tumor local progression and post-treatment tissue changes [14, 15].

The exact time of the early post-ablation DW-MRI changes is extremely variable among different institutes and ranges from the first day to the 8th week after ablation based on various institutional protocols [7, 8, 16–18]. In our study, DWI was performed 24 h after MWA and evaluated as an early predictive factor of future local ablation outcomes.

Many authors have evaluated the usage of DW-MRI for assessment of treatment outcomes and local tumor progression post-thermal ablation of the liver [9] and lung tumors [10]. These studies showed increases in ADC values after treatment which reflects a good predictive value for treatment outcome and local tumor response. The mechanism of the increase in ADC value after treatment of cancer is probably due to the accompanying necrosis which leads to shrinkage of cells as well as decreased water content inside cells with subsequent increased interstitial fluid content and extracellular space [10].

DW-MRI at 24 h after percutaneous lung MWA showed reduced signal intensity with statistically significant increased ADC values of ablated lung lesions in comparison with values before ablation. Also, a significant difference is observed in the post-ablation ADC values of lesions with (1.4 ± 0.3 × 10^−3^ mm^2^/s) and those without local tumor progression (1.7 ± 0.3 × 10^−3^ mm^2^/s, *p* = 0.001). These results suggest that pre-ablation and post-MWA DW-MRI signals with ADC values calculated from the ADC map can be used as an early predictor for treatment outcome before the changes in the tumor morphology detected on CT Images.

Okuma et al [10] studied the role of the diffusion-weighted imaging in the early post-ablation assessment of pulmonary tumors on 17 patients having 20 malignant lung lesions. In the follow-up examinations by MRI done 3 days after radiofrequency ablation (RFA) of pulmonary malignant lesions, they obtained results that were similar to ours regarding the value of ADC measurement following local thermal ablation but they did not report a post-ablation cut-off value to predict response as stated in their protocol.

In another study of MRI after RFA in normal animal lung tissues, the ablation zone appeared isointense in T1 WI and hypointense on T2 WI and this may be attributed to coagulative necrosis following the ablation procedure. The peripheral part of the ablation zone showed a hyperintense signal on T2 WI due to neutrophilic cell infiltration and fluid collections inside lung alveoli, which was proved pathologically in a porcine model [17]. In the current study, the T1 WI and T2 WI signals were going in context with these results. We suggest that the peripheral high signal intensity seen in T2 WI is related to perilesional edematous and inflammatory changes, and this explains that the lesions showed significant marginal rim contrast enhancement in post-ablation contrast-enhanced-T1 WI.

They noticed that the ablated area on T1 WI was isointense before and after the procedure, which masked good assessment of the ablated lesion. They also stated that the low signal area on T2 WI causes underestimation of the ablated lesion size [19].

In the current study, a significant post-ablation increase in ADC values was noticed and post-ablation values of ADC were lower with lesions of future local progression compared to lesions with future local tumor response and we suggested a cut-off ADC value of 1.42 as a reference point for prediction of the tumor response (it had sensitivity of 66.67%, specificity of 84.21%, PPV of 66.7%, and NPV of 84.2%), with lesions of mean ADC value below this point having the probability of future local tumor progression and those of higher ADC values having a probability of showing local tumor response.

A significant relation between pre-ablation tumor size and the response (*p* = 0.018) was noticed. Also, the mean diameter of tumors was significantly larger in lesions with local progression and this is matched with other lung local ablation studies which concluded that tumors with a diameter larger than 3 cm are more likely to have local progression [10, 20–23] and, in our study, we found that local tumor progression is more frequent in tumors more than 2 cm in diameter.

The limitations of the current study include its retrospective nature, short period of follow-up in some patients which may develop tumor progression later, and the difficulty in detecting small lesions less than 5 mm in DWI-MRI, in addition to the susceptibility and motion artifacts from respiratory and cardiac motion. The precise determination of time to tumor progress depends on imaging follow-up and time gaps between follow-ups. In some patients, it was as short as 6 months post ablation. This limiting factor was governed by the institutional tumor board regulations, which define the frequency of follow-ups.

Also, the ROI was put to include most of the ablated tumor; hence, the local progression outside the ROI was not assessed. Also, we included different types of pulmonary tumors including primary and metastatic neoplasms.

In conclusion, the ADC value which is calculated from DW-MRI done 24 h after tumor ablation is a quantitative measurement that may predict the treatment efficacy of percutaneous microwave ablation for the treatment of inoperable lung neoplasms in early post-ablation imaging follow-up before changes in the tumor morphology could be detected on CT scan.

## Abbreviations

ADC: Apparent diffusion coefficient
DWI: Diffusion-weighted imaging
DW-MRI: Diffusion-weighted magnetic resonance imaging
HCC: Hepatocellular carcinoma
INR: International normalizing ratio
MWA: Microwave ablation
RF: Radiofrequency
ROC: Receiver operating curve
ROI: Region of interest

## Declarations

### Guarantor

The scientific guarantor of this publication is Prof. Dr. Thomas J Vogl.

### Statistics and Biometry

No complex statistical methods were necessary for this paper.

### Methodology

• Retrospective

• Observational

• Performed at one institution
